# Inspiratory Muscle Strength in Chagas Cardiomyopathy: A Systematic Scoping Review

**DOI:** 10.1590/0037-8682-0389-2023

**Published:** 2023-12-08

**Authors:** Flavia Mazzoli-Rocha, Clara Pinto Diniz, Daniel Pereira Redes de Rezende, Fernanda de Souza Nogueira Sardinha Mendes, Alejandro Marcel Hasslocher-Moreno, Andréa Silvestre de Sousa, Roberto Magalhães Saraiva, Audrey Borghi-Silva, Mauro Felippe Felix Mediano, Dângelo José de Andrade Alexandre

**Affiliations:** 1 Laboratório de Pesquisa Clínica em Doença de Chagas, Instituto Nacional de Infectologia Evandro Chagas, Fundação Oswaldo Cruz, Rio de Janeiro, RJ, Brasil.; 2 Departamento de Fisioterapia, Instituto Nacional de Traumatologia, Rio de Janeiro, RJ, Brasil.; 3 Laboratório de Fisioterapia Cardiopulmonar, Universidade Federal de São Carlos, São Carlos, SP, Brasil.; 4 Departamento de Ensino e Pesquisa, Instituto Nacional de Cardiologia, Rio de Janeiro, RJ, Brasil.

**Keywords:** Chagas cardiomyopathy, Maximum inspiratory pressure, Inspiratory muscle strength

## Abstract

The increase in inflammatory markers associated with persistent chronic fibrosing myocarditis, a characteristic of chronic Chagas disease, can result in a reduction in inspiratory muscle strength (IMS) in Chagas cardiomyopathy (CC). However, literature in this field is still scarce. This review aimed to map and summarize the evidence regarding IMS in patients with CC. The inclusion criteria included reports with adult participants with a CC diagnosis, with or without heart failure (HF). The core concept examined was the maximum inspiratory pressure evaluated in the untrained and trained groups in the pre-training period. The context was open, including but not limited to hospitals and health centers. Two authors independently identified eligible studies and extracted the data. Descriptive synthesis was used as the primary strategy for analyzing the results. Nine studies (five clinical trials, three cross-sectional, and one cohort) were included. The CC classification differed among the studies, with no mention of HF in five and no CC staging specification in six. IMS was assessed using a manovacuometer, and only six studies analyzed and interpreted the data concerning the predicted values. The CC population with HF appeared to have impaired IMS. All studies involved only Brazilian volunteers. In conclusion, randomized clinical trials evaluating IMS and the effects of inspiratory muscle training need to be conducted to better understand the prevalence and risk of inspiratory muscle weakness in the CC population, as well as the effects of training. Such studies should be conducted at different stages of CC in different populations and countries.

## INTRODUCTION

Chagas disease (CD), a neglected infectious disease, affected more than 5 million people and caused 12,000 deaths in 2010 in 21 endemic countries in Latin America^1^. Despite the implementation of control measures for vector transmission in Latin American countries, CD has spread worldwide and is no longer restricted to endemic areas. Congenital, transfusional, and transplant transmission routes are important in non-endemic countries^2^. The cardiac form of CD, usually known as Chagas cardiomyopathy (CC), is the leading cause of non-ischemic cardiomyopathy in Latin America, affecting 20%-40% of the infected people^3^. The staging of CC is based on electrocardiographic and echocardiographic changes and the presence of heart failure (HF), constituting stages A, B1, B2, C, and D^4^. With a slow and persistent course, the chronic form is characterized by chronic fibrosing myocarditis with myocardial fibers replaced by scar tissue^5^, causing manifestations such as dilated cardiomyopathy, arrhythmias, stroke, and HF^3^. 

The increase in inflammatory markers, usually present in HF, and the persistent chronic fibrosing myocarditis characteristic of CD can cause respiratory muscle weakness^6^ ^-^ ^8^ and loss of the ability to increase respiratory work to meet the peripheral oxygen demand, resulting in reduced exercise capacity^9^ and left ventricular ejection fraction (LVEF)^10^ ^,^ ^11^. In addition, a reduced LVEF in CC seems to increase the risk of inspiratory muscle weakness (IMW)^12^.

Inspiratory muscle strength (IMS) is considered an independent risk factor for worse prognosis in patients with HF from other etiologies, and a maximum inspiratory pressure < 76 cmH_2_O has been associated with a lower 36-month survival^13^. Studies on IMS in CC conducted in both sexes, aged approximately 50 years, and LVEF between 40% and 50%, used maximal inspiratory pressure to assess IMS in patients with CC, with no stratification according to the presence or absence of HF^12^ ^,^ ^14^.

A preliminary search was conducted, and no scoping or systematic review addressing a similar topic was found. Despite the growing number of studies on the topic, a scoping review is appropriate to determine which types of evidence are available. The sociodemographic and clinical characteristics of the studied population, outcomes used, follow-up period, type of devices used, and which health professionals performed the analysis of IMS have been reported in the studies investigating IMS in the CC population. Therefore, this scoping review aimed to map and summarize the evidence on IMS in the CC population and answer six questions: (1) “What types of evidence are available on IMS at different stages of CC?”; (2) “What are the sociodemographic and clinical characteristics of the population in the studies investigating IMS during CC?”; (3) “What outcomes are used in the studies to assess IMS during CC?”; (4) “What are the follow-up periods used in the studies investigating IMS during CC?”; (5) “What devices are used in the studies to analyze IMS?”; and (6) “Which health professionals performed the analysis of IMS?”

## METHODS

### ● Protocol and registration

This scoping review followed the methodological recommendations of the Joanna Briggs Institute (JBI) Manual for Evidence Synthesis^15^ and the writing guidelines of the Preferred Reporting Items for Systematic Review and Meta-Analysis for scoping reviews (PRISMA-ScR)^16^. It was registered with the Open Science Framework on July 6, 2022 (10.17605/OSF.IO/KXJNM).

### ● Eligibility criteria

The eligibility criteria for the study inclusion were based on descriptions of the participants, concepts, contexts, and types of evidence sources^15^ (Table 1). 

**TABLE 1:** Eligibility criteria for the inclusion of studies.

Components	Description
Participants	Adults aged 18 years or older with the diagnosis of Chagas cardiomyopathy, with or without heart failure.
Concept	Maximum inspiratory pressure in the untrained and trained groups in the pre-training moment (baseline data).
Context	Open context, including but not limited to hospitals and health centers.
Types of evidence sources	Primary clinical studies (quantitative, qualitative, and mixed-method design) and review studies reported as full text or abstract. Studies include systematic reviews, scoping reviews, randomized controlled trials, nonrandomized clinical trials, prospective cohorts, retrospective cohorts, case-control, case series, and case reports. Protocols, conference abstracts, editorials, expert opinions, and other non-peer-reviewed documents were excluded. No restrictions on language, year of publication, or publication status.

### ● Information sources and search strategies

One author (DJAA) conducted a search to identify studies in the electronic databases. A search was performed on the MEDLINE/PubMed database, and the primary studies that answered the research questions were retrieved. The search strategy was adjusted and used to build search strategies for the other databases. The World Health Organization (WHO) International Clinical Trials Registry Platform (ICTRP) was consulted for ongoing or unpublished randomized clinical trials. A search for retracted and errated statements relevant to the information from the included studies was performed using the MEDLINE/PubMed and EMBASE/Elsevier. All search strategies are presented in Appendix 1 and follow the Peer Review of Electronic Search Strategies (PRESS) 2015 Guideline Statement^17^. The last search date for each database was February 18, 2023, without restrictions.

### ● Selection of sources of evidence and data charting process

To confirm the understanding of the eligibility criteria and reduce errors in the selection of sources of evidence, calibration was carried out with the authors from 25 randomly chosen studies. Such calibration occurred through a meeting in which agreements and disagreements were verified in the initial selection and doubts were clarified. Adjustment of the eligibility criteria was unnecessary. Achieving 75% or higher agreement among the reviewers was the goal of selecting the studies. To select the sources of evidence, duplicates were identified and excluded. A single main publication was considered for multiple publications from the same study and secondary publications were considered duplicates. Two authors (DPRR and CPD) independently screened the titles and abstracts for the eligibility criteria of the scoping review, and consensually excluded or considered eligible for the next step. The study was considered eligible for the next step in case of doubts regarding eligibility owing to insufficient information from the title and abstract. Finally, the full texts of the eligible studies were retrieved, and two authors (DPRR and CPD) carefully analyzed the eligibility criteria. A list of studies excluded after reading the full texts for at least one explicit reason for exclusion is available in the document attached to the full scoping review (Appendix 2). For the entire selection of sources of evidence, the Rayyan^18^ tool was used, which is displayed according to the flowchart of the PRISMA-ScR^16^.

Bibliometric information, participant characteristics, concepts (evaluated outcomes), contexts, and other relevant information were incorporated into an organized data mapping form. To check whether it was necessary to modify or add any relevant issues, training was conducted with the authors and pilot data were mapped from the first two primary studies and two other review studies among those already included. The data mapping form was adjusted (Appendix 3) and performed independently by two authors (DPRR and CPD). One reviewer did not have access to the other’s responses until the consensus stage. 

Disagreements were resolved by consensus, and if necessary, by consulting a third author^15^ (DJAA).

### ● Synthesis of results

Descriptive synthesis was used as the primary strategy for analyzing the results. Data mapped from the structured form are presented in a tabular format and graphics. The synthesized data were accompanied by a narrative summary that correlated with the scoping review objectives.

## RESULTS

**FIGURE 1:**
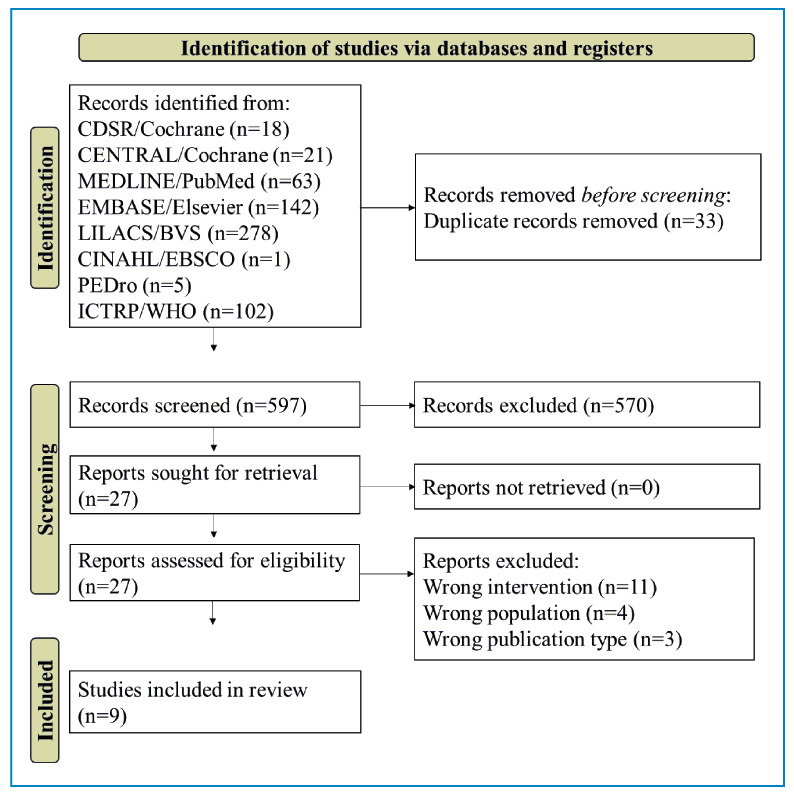
Flow diagram with identification, screening, and included studies.

Figure 1 shows that after removing the duplicate records, 597 records were excluded from screening since they did not meet the eligibility criteria. A total of 27 records were retrieved and assessed for eligibility by reading the full-text records. Of these, 18 were excluded due to inappropriate population, intervention, or publication type. Therefore, nine studies were included in this scoping review. No additional studies were obtained from searches using other methods, such as the references of the included studies or other databases. 

### ● Characteristics of the included studies

The data mapped based on the characteristics of the included studies are presented in Table 2. This scoping review considered all types of studies, including five clinical trials, three cross-sectional studies, and one cohort study. Of the five clinical trials, only three were randomized. Only one study directly related to IMS evaluated the hemodynamic response of two inspiratory muscle training (IMT) protocols. The other two studies evaluated the effects of aerobic and peripheral muscle training and noninvasive ventilation, with IMS as a secondary analyzed variable. However, one study did not mention the location. Additionally, four of the included studies were performed using only one volunteer approach and had no follow-up period. In the other five studies, the follow-up period was related to the cardiac rehabilitation program (CRP) (3), non-invasive ventilation protocol (1), or hemodynamic response to IMT (1).

**TABLE 2:** Characteristics of the included studies.

Articles	Type of study	Context	Follow-up
Baiao et al., 2013	Cross-sectional study	Not informed	No
Begot et al., 2021	Single-arm prospective cohort study	Hospital (Inpatient)	No
Carvalho et al., 2017	Randomized single-blind (assessor) crossover controlled clinical trial	Cardiopulmonary Physical Therapy Laboratory at the Federal University	Yes
Costa et al., 2017	Cross-sectional study	Hospital (Referral Outpatient Center)	No
Mendes et al., 2020	Randomized open-label parallel-group controlled clinical trial	Clinic (Outpatient)	Yes
Frota et al., 2020	Randomized open-label crossover controlled clinical trial	Clinic (Outpatient)	Yes
Mediano et al., 2016	Single-arm clinical trial	Clinic (Outpatient)	Yes
Mendes et al., 2011	Non-randomized open-label parallel-group controlled clinical trial	Laboratório de Doença de Chagas da Universidade Estadual de Maringá	Yes
Vieira et al., 2014	Cross-sectional study	Clinic (Outpatient)	No

### ● Sociodemographic and clinical characteristics of the population of the included studies

The data mapped from the sociodemographic and clinical characteristics of the study population of the included studies are presented in Table 3. The participants in all nine studies were older than 18 years. Eight of these studies included both sexes.

**TABLE 3:** Sociodemographic and clinical characteristics of the population of the included studies.

Articles	Sample size	Age (year)	BMI (kg/m^2^)	Stage of CC	Presence of HF	NYHA	LVEF (%)	Functional capacity (ml/kg/min or meters)	Period of study	MIP (cmH_2_O)
Baiao et al., 2013	15 (46.7)	50.3 (5.7)	24.93 (4.03)	-	-	II: 87% (13)	36.00 (34.45-2.88)	28.46 (6.58)^#^	-	50.0 (46.7-76.0)
						III: 13% (2)				
Begot et al., 2021	21 (28.6)*	50.1 (10.8)*	-	-	100% (21)*	III and IV*	26.09 (7.70)*	255.93 (80.69)*^&^	2013-17	77.6 (25.1)*
Carvalho et al., 2017	24 (33.3)	51.8 (10.2)*	27.1 (4.0)*	-	100% (24)*	II: 62.5% (15)*	36.0 (8.4)*	18.3 (4.4)*	-	70.2 (23.9)*
						III: 37.5% (9)*				
Costa et al., 2017	48 (70.8)	56.4 (53.3-59.5)	-	-	-	I: 56% (27)	54.3 (48.6-59.9)	20.4 (18.8-22.0)	2015-16	35% (17) of IMW
						II: 36% (17)				
						III: 8% (4)				
Mendes et al., 2020	30 (33.3)	59.0 (10.0)	BC: 25.6 (4.3)	B2: 27% (8)	73.3% (22)	-	33 (8)	BC = 15.4 (6.3)	2015-17	BE = 81.1 (22.9)
			BE: 25.1 (6.2)	C: 73% (22)				BE = 17.6 (4.7)		BC = 74.2 (33.6)
Frota et al., 2020	21 (42.9)	60.3 (11.9)	27.0 (3.4)	B2: 33.3% (7)	66.7% (14)	-	40.4 (10.1)	-	-	64.0 (31.6)
				C: 66.7% (14)						
Mediano et al., 2016	12 (75.0)	56.1 (13.8)	25.5 (4.2)	C: 83.3% (10)	100% (12)	I: 25% (3)	31.9 (7.7)	15.8 (5.2)	2013-14	62.3 (26.6)
				D: 16.7% (2)		II: 58.3% (7)				
						III: 16.7% (2)				
Mendes et al., 2011	14 (100.0)	BE = 48.0 (5.0)	-	-	-	I: 100% (14)	-	BC = 22.2 (6.3)	2007-08	BE = 80.0 (5.0)
		BC = 53.6 (6.2)						BE = 31.1 (4.3)		BC = 80.9 (9.9)
Vieira et al., 2014	16 (43.8)	53.5 (9.2)	23.3 (4.1)	-	-	I: 18.8% (3)	34.1 (8.0)	481.8 (109.5)^&^	2010	51.6 (25.8)
						II: 56.2% (9)				
						III: 25% (4)				

Sample size values were expressed as absolute numbers (percentages of females). Age, BMI, LVEF, functional capacity, and MIP are presented as mean (SD) or median (interquartile range). Values for the CC stage, presence of HF, and NYHA classification are presented as percentages (absolute numbers). BMI, body mass index; CC, Chagas cardiomyopathy; HF, heart failure; NYHA, New York Heart Association functional classification of heart failure; LVEF, left ventricular ejection fraction; MIP, maximum inspiratory pressure; BE, baseline exercise group; BC, baseline control group; IMW, inspiratory muscle weakness. * Data from all study patients, including patients with heart failure from Chagas disease and other etiologies. ^#^ Estimated VO_2_. ^&^ Distance traveled in the Six Minute Walk test in meters.

All included articles exclusively studied CC-diagnosed patients, except for two of them^19^ ^,^ ^20^, in which CC patients represented only 40% and 17% of all samples, respectively. To assess the severity of cardiac disease, studies have used the CC stage (3), the presence of HF (5), or NYHA (7). Of the nine studies, HF was 100% present in patients included in three and predominantly (> 65%) in two. Four studies did not mention the presence or absence of HF in patients, and only three used the CC stage classification. The NYHA criterion was used for almost all patients. LVEF was calculated in all but one study. The mean was 34.9%, considering articles that presented LVEF as the mean and involved only patients with CC. 

To analyze the functional capacity, five studies used peak oxygen uptake (VO_2_peak) assessed through cardiopulmonary exercise testing (CPET). The other protocols used the estimated oxygen uptake expressed as VO_2_ and the distance reached in the 6-minute walk test. Only one study did not analyze functional capacity. The mean of the means of the VO_2_peak was 20 ml/kg/min. As expected, all articles measured the maximum inspiratory pressure (MIP), presented as mean or median, with mean values ranging from 51.6 to 81.1 cmH_2_O. Of the nine records, three evaluated only the absolute value of MIP, without analysis or interpretation of data concerning the predicted values. In the other six records, the MIP data were controversial, but they generally suggested a reduction in IMS in patients with CC and HF.

All nine studies were conducted in Brazil over the last 16 years. MIP was assessed by a manovacuometer device in all of them. Inspiratory muscle endurance and physical activity levels were evaluated in only one study. Electrocardiogram, wall motion abnormality (echocardiogram), dyspnea, and peripheral muscle strength were not mentioned in any study. There was no mention of the professional who performed the MIP assessment or whether adverse events occurred.

## DISCUSSION

### ● Main findings

Our scoping review of the studies investigating the CC population and the IMS identified six key findings relevant to our research question:

1. The unique clinical trial investigating the CC population directly related to IMS evaluated the hemodynamic responses of two IMT protocols.
2. All included studies involved only Brazilian volunteers.
3. The CC classification differed among the studies, with no mention of the presence of HF in five studies and no CC staging specification in six studies.
4. Only one study evaluated inspiratory muscle endurance, and three evaluated MIP as an absolute value without analysis and interpretation of data related to the predicted values.
5. Five studies were done with a follow-up period, but none related to the effects of IMT on the IMS of the CC population.
6. In all nine studies, no mention was made concerning which health professionals performed the analysis and interpretation of IMS data.

### ● Comparison to the current state

The evaluation of IMS and the inclusion of IMT in cardiac rehabilitation programs (CRP) are well-defined once the benefits of IMT have been demonstrated in patients with HF. In a systematic review and meta-analysis, Azambuja et al.^21^ indicated that isolated IMT increased IMS, functional capacity, and quality of life (QoL), which were more pronounced in patients with IMW. However, this evidence of benefits has not yet been confirmed in the CC population. In this scoping review, three randomized clinical trial studies were included related to the effect of aerobic training with or without peripheral muscle training in CRP^21^ ^-^ ^24^, non-invasive ventilation^20^, and hemodynamic responses of two IMT protocols^25^. Interestingly, of the three studies that investigated the effects of CRP, only one analyzed the MIP values with respect to the predicted values. In general, the MIP value seems to be an isolated variable, without a specific purpose in these studies. Moreover, no study has evaluated the effects of IMT in patients with reduced IMS or IMT on lung function, functional capacity, or QoL in the CC population. 

Currently included in the group of neglected tropical diseases by the World Health Organization (WHO)^26^, Chagas has become a worldwide public health concern due to the unknown, unrecognized, and untreated seropositive population immigrating to non-endemic areas^1^ ^,^ ^27^. Since it was first described more than 100 years ago, there remains a lack of studies, resources, and strategies to improve the diagnostic methods and the etiological and non-pharmacological treatment of the chronic phase, including CRP and the role of IMT in CRP^28^. Moreover, despite effective therapies, approximately 70%-90% of individuals affected by CD are unaware of their diagnosis^1^. Additionally, most cases of CC occur in the Latin American and Caribbean regions^30^. Although several people with *T. cruzi* infection are increasingly recognized in the United States and Europe^27^ ^,^ ^31^, all the studies in this scoping review were conducted in Brazil. In line with this, Brazil has advanced non-pharmacological treatment of CD^23^ ^,^ ^24^ ^,^ ^32^, illustrating the need for further research in this area. However, it is important to note that the genetic variability of the parasite and host may result in different results if studies are conducted in different geographical regions.

Another issue to be highlighted is the need to standardize the samples studied concerning the stage of CC by using a classification of cardiac involvement and disease severity to facilitate comparison among clinical studies and their external validation. Although there are different classifications for CC^3^, the Brazilian Consensus on Chagas Disease (2015)^4^ suggests staging myocardial impairment into five distinct subgroups with prognostic value, as suggested by Xavier et al. (2005)^33^. In this classification, CC is stratified as: A (normal LVEF, absence of segmental alterations); B1 (mild systolic dysfunction, with LVEF ≥ 45%, and/or presence of segmental alterations); B2 (LVEF < 45%, with or without segmental alterations); C (compensated HF); and D (refractory HF). In this scoping review, only three authors used this classification and only four studies specified the presence of HF. In addition to heart diseases of other etiologies, CC may present HF as the final path, considering a multifactorial systemic disease involving adaptive changes in the cardiovascular, renal, neuroendocrine, hemostatic, immune, inflammatory, and musculoskeletal systems to meet physiological demands^34^. These difficulties in the clinical diagnosis variation provides a simple overview of the CC population.

Some studies have suggested that skeletal muscle deterioration may be a component of HF symptoms, and IMW seems to occur in a greater proportion than peripheral muscle weakness in the HF population^35^ ^,^ ^36^. Specifically, HF with CD etiology seems to have an aggravating factor in the pathophysiology of IMW since there is an additional intense inflammatory process that can induce myositis in the acute and chronic phases, resulting in the degeneration and necrosis of muscle fibers, leading to reduced muscle contractility^37^ ^,^ ^38^. The immune response in the skeletal muscle, secondary to parasite tropism for this tissue, is more intense in the acute phase, but parasites are not completely cleared in the chronic phase^39^. In HF of other etiologies, IMW was associated with worse symptoms, such as dyspnea and fatigue, impaired exercise capacity, QoL, and survival^13^ ^,^ ^36^ ^,^ ^40^. Moreover, IMS is important for determining the prognosis and therapeutic options for individuals with HF^36^ ^,^ ^41^, supporting non-pharmacological treatment. These approaches have improved respiratory functions, such as MIP, MEP, resistance, exercise capacity, and QoL, especially in combined inspiratory muscle and aerobic training protocols^42^ ^,^ ^43^. In a systematic review with meta-analysis investigating the effects of IMT in patients with HF, with 13 randomized controlled trials, isolated IMT resulted in an increase in MIP, functional capacity, and QoL. This improvement was greater in patients with IMW, a training load higher than 60% of MIP, and longer periods of intervention^21^. In this scoping review, only one study^12^ investigated the predictors of IMW in the CC population. The authors observed an increased risk of IMW in subjects with a sedentary lifestyle, lower LVEF, and higher ventilatory inefficiency, suggesting that these are independent risk factors for CC. Considering the benefits of IMT in patients with HF of other etiologies, studies on the effects of IMT in CC populations are urgently needed. 

The IMS is applied to different populations, ranging from healthy athletes to individuals with chronic diseases and metabolic alterations. In these patients, it is important to measure the severity of muscle involvement, screen and diagnose weakness, monitor the course of the disease, evaluate the effectiveness of treatment, and follow-up patients with CRP^44^. This parameter can be helpful in clinical practice and research when interpreted in conjunction with other respiratory functional measures, such as lung function tests and clinical signs and symptoms^44^ ^,^ ^45^. Inspiratory muscle dysfunction can occur due to the loss of at least one of its properties, muscle strength, and endurance, or they may coexist, possibly reflecting chronic increased ventilatory demand^46^. Defined as the capacity to maintain a specific muscular task over time, endurance has already been described and used in clinical practice^47^, and is reported to be lower in patients with HF^40^. Only one study has assessed this parameter in patients with HF and CC patients^14^ and has found reduced values. 

There are a number of ways to assess IMS, but the most used in clinical practice are voluntary tests of respiratory muscle strength, which estimate the global static strength of the inspiratory and expiratory muscles, measuring mouth pressure in a simple way that is well-tolerated by patients^48^. The maximum static inspiratory pressure measured by manovacuometry is widely used in clinical practice. It must be applied by an experienced professional who must strongly urge subjects to perform a maximum inspiratory isometric effort from the residual volume and sustain it for 1-2 s (Mueller maneuver) with a mouthpiece through an occluded valve, featuring a quasi-static maneuver^48^. This type of measurement may be influenced by the chest wall, lung volume, and skeletal muscle length, which reflect muscle strength during isometric contractions in a specific position of the respiratory muscle fibers^45^. All nine studies included in this review used manovacuometry, and no study has dynamically evaluated IMS in patients with CC. 

Careful instruction and encouraged motivation are essential, and individuals must be trained to prevent air leakage through the nozzle, and consequently, depressurization of the system and wrong measurement^45^. In Brazil, manovacuometry is widely used in respiratory physiotherapy, and professionals are the most trained and familiar with this technique in different clinical practice scenarios. Although the IMS was evaluated in all nine studies in this scoping review, only five had a follow-up in the design. Of these, two evaluated the IMS only at baseline for sample characterization, and the other three used the IMS as one variable to assess the effect of CRP. However, no specific intervention for IMT has been proposed. Although they presented different follow-up periods with different interventions, their results were conflicting. Mediano et al.^23^, in a single-arm trial with 8 months of follow-up of CRP with aerobic and peripheral training, and Mendes et al.^22^ in a non-randomized open-label parallel-group clinical trial with 6 weeks of follow-up of CRP with aerobic training, found a significant increase in IMS after the intervention. Mendes et al.^24^, in a randomized open-label parallel-group clinical trial with six months of follow-up for CRP with aerobic and peripheral muscle training, found no significant difference in IMS after the intervention. None of these articles compared IMS with the predicted value for the evaluated population without rating the IMW, making it difficult to understand the clinical relevance of the statistical differences found. Comparison with the predicted value is also important for identifying the prevalence of IMW in patients with CD. This prevalence was only evaluated by Frota et al.^25^ who observed 47.6% IMW in CC patients with HF, as reported by Nakagawa et al.^36^ found in patients with HF of other etiologies. There remains a gap regarding the prevalence of IMW in patients with CC at different stages of the disease, particularly before the progression to HF.

### ● Towards scientific evidence related to inspiratory muscle strength in the CC population

**FIGURE 2:**
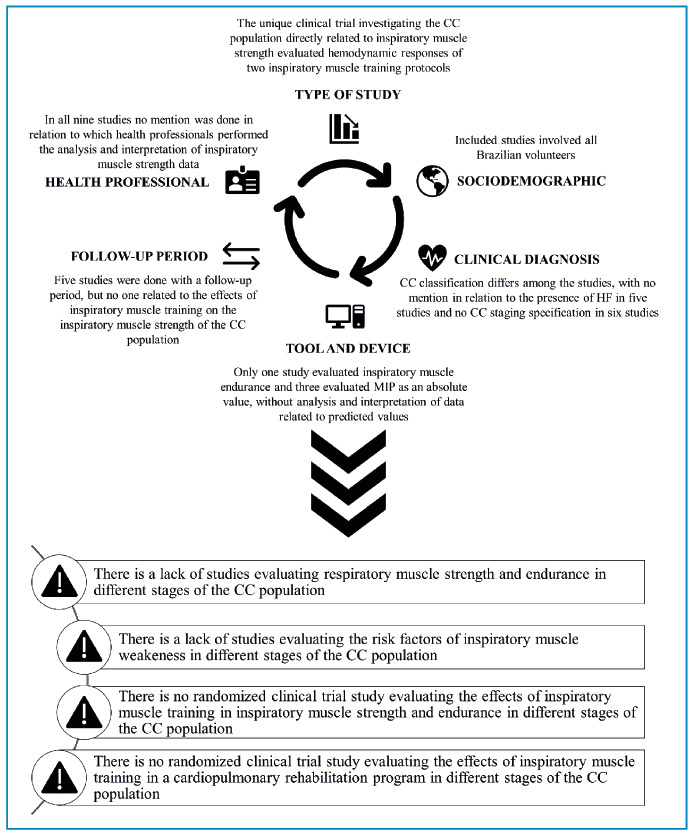
Towards scientific evidence related to inspiratory muscle strength in the CC population.

The main findings extracted from this scoping review are summarized in Figure 2, which highlights the specific aspects of inspiratory muscle issues in patients with CC. An advantage of identifying the gaps in the scientific literature is that it enables paths to be followed by subsequent research towards scientific evidence. Moreover, it may improve the CRP levels in the public health system for this population with neglected diseases. 

Owing to financial and time limitations, we did not assess the risk of bias or certainty of evidence from the included studies. Given the purpose of the scoping review, the Joanna Briggs Institute (JBI) Manual for Evidence Synthesis^15^ does not consider these steps mandatory.

## CONCLUSIONS

Randomized clinical trials evaluating IMS and the effects of inspiratory muscle training should be conducted to better understand the prevalence and risk of inspiratory muscle weakness factors in the CC population, as well as the effects of training. Additionally, these studies should be conducted at different stages of the CC population and in different countries.
