# Supplementary material for: Inspiratory Muscle Strength in Chagas Cardiomyopathy: A Systematic Scoping Review
Source: Rev Soc Bras Med Trop. 2023 Dec 8;56:e0389-2023. doi: 10.1590/0037-8682-0389-2023 (PMC10706025; doi:10.1590/0037-8682-0389-2023)
Supplement: Supplementary file 1 [file 1678-9849-rsbmt-56-e0389-2023-supp1.pdf]

APPENDIX 1: SEARCH STRATEGIES

MEDLINE/PUBMED

#1: Chagas Disease[Mesh] OR American Trypanosomiasis OR Trypanosomiasis, American OR Trypanosomiasis, South American OR South American Trypanosomiasis OR Trypanosoma cruzi Infection OR Infection, Trypanosoma cruzi OR Infections, Trypanosoma cruzi OR Trypanosoma cruzi Infections OR Chagas' Disease

#2: Chagas Cardiomyopathy[Mesh] OR Cardiomyopathy, Chagas OR Trypanosomiasis, Cardiovascular OR Cardiovascular Trypanosomiasis OR Chagas' Cardiomyopathy OR Cardiomyopathy, Chagas' OR Myocarditis, Chagas

#3: Chagas Heart Disease OR CHD

#4: #1 OR #2 OR #3

#5: Maximal Respiratory Pressures[Mesh] OR Pressure, Maximal Respiratory OR Pressures, Maximal Respiratory OR Respiratory Pressure, Maximal OR Respiratory Pressures, Maximal OR Maximal Respiratory Pressure OR Maximum Respiratory Pressure OR Maximum Respiratory Pressures OR Pressure, Maximum Respiratory OR Pressures, Maximum Respiratory OR Respiratory Pressure, Maximum OR Respiratory Pressures, Maximum OR Maximal Inspiratory Pressure OR Inspiratory Pressure, Maximal OR Inspiratory Pressures, Maximal OR Maximal Inspiratory Pressures OR Pressure, Maximal Inspiratory OR Pressures, Maximal Inspiratory OR Maximum Inspiratory Pressure OR Inspiratory Pressure, Maximum OR Inspiratory Pressures, Maximum OR Maximum Inspiratory Pressures OR Pressure, Maximum Inspiratory OR Pressures, Maximum Inspiratory OR Maximal Expiratory Pressure OR Expiratory Pressure, Maximal OR Expiratory Pressures, Maximal OR Maximal Expiratory Pressures OR Pressure, Maximal Expiratory OR Pressures, Maximal Expiratory OR Maximum Expiratory Pressure OR Expiratory Pressure, Maximum OR Expiratory Pressures, Maximum OR Maximum Expiratory Pressures OR Pressure, Maximum Expiratory OR Pressures, Maximum Expiratory

#6: MIP OR Inspiratory Muscle Weakness OR IMW OR Inspiratory Muscle Strength OR IMS OR Respiratory Muscle Strength OR Respiratory Muscle Weakness

#7: #5 OR #6

#8: #4 AND #7

CINAHL/EBSCO

("Chagas Disease"[Mesh] OR "Chagas Disease" OR "Chagas Heart Disease" OR "Chagas Cardiomyopathy")

AND

("Maximal Respiratory Pressures"[MeSH Terms] OR "Maximal Respiratory Pressure" OR "Inspiratory Muscle Weakness" OR "Inspiratory Muscle Strength" OR "Respiratory Muscle Strength" OR "Respiratory Muscle Weakness")

EMBASE/ELSEVIER

#1: 'Chagas disease'/exp

#2: (Chagas Disease) OR (American trypanosomiasis) OR (Chagas infection) OR (Chagas mazza disease) OR (disease, Chagas) OR (infection by Trypanosoma cruzi) OR (infection of Trypanosoma cruzi) OR (south american trypanosomiasis) OR (Trypanosoma cruzi infection) OR (trypanosomiasis, American) OR (trypanosomiasis, South American)

#3: 'Chagas cardiomyopathy'/exp

#4: (cardiomyopathy in Chagas disease) OR (Chagas disease cardiomyopathy) OR (Chagas heart disease) OR (Chagas myocardiopathy) OR (Chagas myocarditis) OR (chronic Chagas disease cardiomyopathy)

#5: (Chagas Heart Disease) OR (CHD)

#6: #1 OR #2 OR #3 OR #4 OR #5

#7: 'maximal respiratory pressure'/exp

#8: (maximal respiratory pressures)

#9: (MIP) OR (Inspiratory Muscle Weakness) OR (IMW) OR (Inspiratory Muscle Strength) OR (IMS) OR (Respiratory Muscle Strength) OR (Respiratory Muscle Weakness)

#10: #8 OR #9

#11: #6 AND #10

APPENDIX 1: SEARCH STRATEGIES

CENTRAL/COCHRANE AND CDSR/COCHRANE

#1: [mh "Chagas Disease"]

#2: (Chagas' Disease) OR (Trypanosomiasis, American) OR (Trypanosomiasis, South American) OR (American Trypanosomiasis) OR (Infections, Trypanosoma cruzi) OR (South American Trypanosomiasis) OR (Trypanosoma cruzi Infections) OR (Trypanosoma cruzi Infection) OR (Infection, Trypanosoma cruzi)

#3: [mh "Chagas Cardiomyopathy"]

#4: (Cardiomyopathy, Chagas') OR (Cardiovascular Trypanosomiasis) OR (Chagas' Cardiomyopathy) OR (Trypanosomiasis, Cardiovascular) OR (Cardiomyopathy, Chagas) OR (Myocarditis, Chagas)

#5: (Chagas Heart Disease) OR (CHD)

#6: #1 OR #2 OR #3 OR #4 OR #5

#7: [mh “Maximal Respiratory Pressures”]

#8: (“Maximal Respiratory Pressure”) OR (Maximum Inspiratory Pressures) OR (Inspiratory Pressures, Maximal) OR (Inspiratory Pressure, Maximal) OR (Pressures, Maximum Inspiratory) OR (Maximum Inspiratory Pressure) OR (Inspiratory Pressures, Maximum) OR (Pressure, Maximal Inspiratory) OR (Inspiratory Pressure, Maximum) OR (Maximal Inspiratory Pressures) OR (Maximal Inspiratory Pressure) OR (Pressure, Maximum Inspiratory) OR (Pressures, Maximal Inspiratory) OR (Maximum Expiratory Pressure) OR (Maximum Expiratory Pressures) OR (Maximal Expiratory Pressures) OR (Expiratory Pressure, Maximum) OR (Pressures, Maximum Expiratory) OR (Expiratory Pressures, Maximal) OR (Expiratory Pressures, Maximum) OR (Pressure, Maximal Expiratory) OR (Expiratory Pressure, Maximal) OR (Pressure, Maximum Expiratory) OR (Pressures, Maximal Expiratory) OR (Maximal Expiratory Pressure) OR (Maximum Respiratory Pressure) OR (Pressure, Maximum Respiratory) OR (Pressure, Maximal Respiratory) OR (Pressures, Maximal Respiratory) OR (Pressures, Maximum Respiratory) OR (Respiratory Pressures, Maximum) OR (Maximal Respiratory Pressure) OR (Maximum Respiratory Pressures) OR (Respiratory Pressures, Maximal) OR (Respiratory Pressure, Maximal) OR (Respiratory Pressure, Maximum)

#9: (MIP) OR (Inspiratory Muscle Weakness) OR (IMW) OR (Inspiratory Muscle Strength) OR (IMS) OR (Respiratory Muscle Strength) OR (Respiratory Muscle Weakness)

#10: #7 OR #8 OR #9

#11: #6 AND #10

LILACS/BVS

(mh:"Chagas Disease") OR (American Trypanosomiasis) OR (Chagas' Disease) OR (Infection, Trypanosoma cruzi) OR (Infections, Trypanosoma cruzi) OR (South American Trypanosomiasis) OR (Trypanosoma cruzi Infection) OR (Trypanosoma cruzi Infections) OR (Trypanosomiasis, American) OR (Trypanosomiasis, South American) OR (mh:"Chagas Cardiomyopathy") OR (Cardiomyopathy, Chagas) OR (Cardiomyopathy, Chagas') OR (Cardiovascular Trypanosomiasis) OR (Chagas' Cardiomyopathy) OR (Myocarditis, Chagas) OR (Trypanosomiasis, Cardiovascular) OR (Chagas Heart Disease) OR (CHD)

AND

(mh:"Maximal Respiratory Pressures") OR (Expiratory Pressure, Maximal) OR (Expiratory Pressure, Maximum) OR (Expiratory Pressures, Maximal) OR (Expiratory Pressures, Maximum) OR (Inspiratory Pressure, Maximal) OR (Inspiratory Pressure, Maximum) OR (Inspiratory Pressures, Maximal) OR (Inspiratory Pressures, Maximum) OR (Maximal Expiratory Pressure) OR (Maximal Expiratory Pressures) OR (Maximal Inspiratory Pressure) OR (Maximal Inspiratory Pressures) OR (Maximal Respiratory Pressure) OR (Maximum Expiratory Pressure) OR (Maximum Expiratory Pressures) OR (Maximum Inspiratory Pressure) OR (Maximum Inspiratory Pressures) OR (Maximum Respiratory Pressure) OR (Maximum Respiratory Pressures) OR (Pressure, Maximal Expiratory) OR (Pressure, Maximal Inspiratory) OR (Pressure, Maximal Respiratory) OR (Pressure, Maximum Expiratory) OR (Pressure, Maximum Inspiratory) OR (Pressure, Maximum Respiratory) OR (Pressures, Maximal Expiratory) OR (Pressures, Maximal Inspiratory) OR (Pressures, Maximal Respiratory) OR (Pressures, Maximum Expiratory) OR (Pressures, Maximum Inspiratory) OR (Pressures, Maximum Respiratory) OR (Respiratory Pressure, Maximal) OR (Respiratory Pressure, Maximum) OR (Respiratory Pressures, Maximal) OR (Respiratory Pressures, Maximum) OR (MIP) OR (Inspiratory Muscle Weakness) OR (IMW) OR (Inspiratory Muscle Strength) OR (IMS) OR (Respiratory Muscle Strength) OR (Respiratory Muscle Weakness)

PEDro

Chagas Disease

Chagas Cardiomyopathy

Chagas Heart Disease

APPENDIX 1: SEARCH STRATEGIES

ICTRP/WHO

Chagas Disease

Chagas Cardiomyopathy

Chagas Heart Disease
