# Supplementary material for: Inspiratory Muscle Strength in Chagas Cardiomyopathy: A Systematic Scoping Review
Source: Rev Soc Bras Med Trop. 2023 Dec 8;56:e0389-2023. doi: 10.1590/0037-8682-0389-2023 (PMC10706025; doi:10.1590/0037-8682-0389-2023)
Supplement: Supplementary file 2 [file 1678-9849-rsbmt-56-e0389-2023-supp2.pdf]

APPENDIX 2: STUDIES EXCLUDED AFTER READING THE FULL TEXTS

	Article title	Exclusion reason
1	Relationship between left ventricular ejection fraction and e /e′ ratiowith functional capacity in chagas heart disease	Wrong intervention
2	Cardiac rehabilitation for people with heart disease: an overview of Cochrane systematic reviews	Wrong population
3	Exercise training improves microvascular function in patients with Chagas heart disease: Data from the PEACH study	Wrong intervention
4	Frailty Among Non-Elderly Patients Undergoing Cardiac Surgery	Wrong population
5	Reduced functional capacity in patients with Chagas disease: a systematic review with meta-analysis	Wrong intervention
6	Exercise-induced ventricular arrhythmias and vagal dysfunction in Chagas disease patients with no apparent cardiac involvement	Wrong intervention
7	High intensity intermittent training as an alternative in cardiovascular rehabilitation: a meta-analysis	Wrong population
8	Analysis of functional profile of acute chagas disease patients treated at university hospital	Wrong publication type
9	A randomized trial of the effects of exercise training in Chagas cardiomyopathy	Wrong intervention
10	Assessment of Autonomic Function by Phase Rectification of RRInterval Histogram Analysis in Chagas Disease	Wrong intervention
11	Effects of exercise training on heart rate variability in Chagas heart disease	Wrong intervention
12	Health-related quality of life in patients with Chagas disease	Wrong intervention
13	Bioenergetic and cardiovascular responses to exercise in residents at 2.850 m, with asymptomatic Chagas' disease	Wrong intervention
14	Effect of exercise training on cardiovascular autonomic and muscular function in subclinical Chagas cardiomyopathy: a randomized controlled trial	Wrong intervention
15	Effects of cardiomegaly on regional chest wall volume in patients with chronic Chagas cardiomyopathy	Wrong publication type
16	Measurement of plasma B-type natriuretic peptide (BNP) in patients with chronic Chagas' disease before and after physical exercise program. Is BNP a good marker of better tolerance to physical activity?	Wrong publication type
17	Health-related quality of life in patients with Chagas disease: a review of the evidence	Wrong intervention
18	Evaluating respiratory musculature, quality of life, anxiety, and depression among patients with indeterminate chronic Chagas disease and symptoms of pulmonary hypertension	Wrong population
