# Supplementary material for: Inspiratory Muscle Strength in Chagas Cardiomyopathy: A Systematic Scoping Review
Source: Rev Soc Bras Med Trop. 2023 Dec 8;56:e0389-2023. doi: 10.1590/0037-8682-0389-2023 (PMC10706025; doi:10.1590/0037-8682-0389-2023)
Supplement: Supplementary file 3 [file 1678-9849-rsbmt-56-e0389-2023-supp3.pdf]

APPENDIX 3: DATA MAPPING FORM

Template study details and characteristics and results extraction instrument

Review title: Inspiratory muscle strength in Chagas Cardiomyopathy: A Systematic Scoping Review

Eligibility criteria (PCCS):

Item	Variable
**Age**	Age
**Diagnostic of Chagas disease form**	CC
**Inspiratory muscle strength**	MIP
	S-index
	Inspiratory muscle resistance
	Other
**Location of Study**	Hospitals, health centers, and other
**Type of Study**	Review, clinical trial, observational, and other

CC: Chagas cardiomyopathy; **MIP:** maximum inspiratory pressure.

Study details and characteristics extraction:

Item	Information/Value
**Number of participants**	
**Sex (M or F)**	
**Body mass index**	
**Stage of CC**	A, B1, B2, C or D
	Presence or not of HF
**ECG**	Any alteration
**ECO**	LVEF
	Wall motion abnormality
**Medication**	
**Dyspnea**	Borg
	Other
**Functional capacity**	CPET - VO_2max
	SMWT - Distance
	Other
**Time of study (year)**	
**Location (country)**	
**Professional who performed the analysis of IMS**	
**Device used to measure IMS**	
**Test protocol: force or resistance?**	
**Test protocol: charge**	
**Test protocol: time or number of repetitions**	
**Adverse events during the IMS test**	
**Adverse events after the IMS test**	
**Value of IMS was used to indicate any training?**	
**If yes, were volunteers indicated to do any aerobic training, PMT or IMT?**	
**Dynamotry**	
**Physical activity level IPAQ or similar**	
**Practice of regular physical activity**	
**Occupational activity or retirement**	

M: masculine; F: feminine; **CC:** Chagas cardiomyopathy; **HF:** heart failure; **ECG:** electrocardiogram; **ECO:** echocardiography; **LVEF:** left ventricular ejection fraction; **CPET:** cardiopulmonary exercise test; **VO2max:** maximum consumption of oxygen; **SMWT:** six-minute walking test; **IMS:** inspiratory muscle strength; **PMT:** peripheral muscle training; **IMT:** inspiratory muscle training; **IPAQ:** International Physical Activity Questionnaire.
